# An increase in long non-coding RNA PANDAR is associated with poor prognosis in clear cell renal cell carcinoma

**DOI:** 10.1186/s12885-017-3339-9

**Published:** 2017-05-25

**Authors:** Yi Xu, Yanyue Tong, Jianyong Zhu, Zhangming Lei, Lijun Wan, Xiuwen Zhu, Feng Ye, Liping Xie

**Affiliations:** 1 Department of Urology, Quzhou Hospital, Zhejiang University, Quzhou City, 324000 China; 2 Department of Urology, First Affiliated Hospital, School of Medicine, Zhejiang University, Hangzhou City, 310003 China

**Keywords:** ccRCC, lncRNA, PANDAR, PI3K/Akt/mTOR, Apoptosis

## Abstract

**Background:**

Nearly 30% of clear cell renal cell carcinoma (ccRCC) patients present with metastasis at the time of diagnosis, and the prognosis for these patients is poor. Therefore, novel potential prognostic biomarkers and therapeutic targets for ccRCC could be helpful. Emerging evidence indicates that lncRNAs play important roles in cancer tumorigenesis and could be used as potential biomarkers or therapeutic targets. PANDAR (promoter of CDKN1A antisense DNA damage activated RNA) is a relatively novel lncRNA that plays an important role in the development of multiple cancers. However, the clinical significance and molecular mechanism of PANDAR in ccRCC are still elusive. In the present study, we attempted to elucidate the role of PANDAR in ccRCC.

**Methods:**

The relative expression level of lncRNA PANDAR was quantified by real-time qPCR in 62 paired ccRCC tissues and in renal cancer cell lines, and its association with overall survival was assessed by statistical analysis. The biological functions of lncRNA PANDAR on ccRCC cells were determined both in vitro and in vivo.

**Results:**

PANDAR expression was significantly upregulated in tumor tissues and cell lines compared with normal counterparts. Moreover, PANDAR served as an independent predictor of overall survival, and increased PANDAR expression was positively correlated with an advanced TNM stage. Further experiments demonstrated that PANDAR silencing can significantly inhibit cell proliferation and invasion, induce cell cycle arrest in the G1 phase and significantly promote apoptosis in 7860 and Caki-1 cell lines. In addition, in vivo experiments confirmed that downregulation of PANDAR inhibited the tumorigenic ability of 7860 cells in nude mice. Silencing of PANDAR also inhibited the expression of Bcl-2 and Mcl-1 and upregulated the expression of Bax in vivo.

**Conclusions:**

Our results suggest that PANDAR is involved in ccRCC progression and may serve as a potential prognostic biomarker and therapeutic target.

## Background

Renal cell carcinoma (RCC) accounts for approximately 3% of all malignancies and represents the most lethal urological cancer with approximately 202,000 cases and 102,000 deaths worldwide [1, 2]. Clear cell renal cell carcinoma (ccRCC) is the most common subtype of RCC and is responsible for nearly 85% of all RCC cases [1]. The wide application of ultrasound and computed tomography has shown that about one-third of ccRCC patients with newly diagnosed disease show evidence of metastases that are associated with a poor prognosis, and the median survival time for these patients is only 13 months [3]. Despite numerous studies that have shown that many genetic and epigenetic changes are associated with the development and progression of ccRCC, the molecular mechanism of renal cancer pathogenesis is still elusive, and the prognosis remains poor. Therefore, the identification of sensitive and specific ccRCC targets and the development of novel therapeutic strategies is urgently needed.

Long noncoding RNAs (lncRNAs) are a newly discovered class of noncoding RNAs (ncRNA) that are longer than 200 nucleotides and are not translated into proteins [4]. Mounting evidence has indicated that lncRNAs play important roles in diverse biological processes, such as cell growth, cell death, stem cell pluripotency, tumorigenesis and development [5]. The rapid development of high-throughput RNA sequencing and cancer genomics also highlighted the significance of lncRNA in various human cancers [6, 7]. However, the molecular mechanism and clinical significance of lncRNA in ccRCC remains largely unknown.

PANDAR (promoter of CDKN1A antisense DNA damage activated RNA) is a relatively new lncRNA that is localized at 6p21.2 [8]. PANDAR is induced after DNA damage in a p53-dependent pattern, and it interacts with the transcription factor NF-YA to repress the expression of pro-apoptotic genes [8]. Both DNA damage and NF-YA are closely associated with tumorigenesis [9, 10]. Therefore, PANDAR may play an important role in the development of cancers. Recently, it has been reported that the expression of PANDAR was downregulated in non-small cell lung cancer (NSCLC) and a low level of PANDAR was associated with a poor prognosis [11]. In contrast, PANDAR was found to be upregulated in hepatocellular and in bladder carcinoma, and a high level of PANDAR was associated with a poor prognosis [12]. These studies indicate that PANDAR plays controversial roles in cancers. Moreover, the role of PANDAR in ccRCC has not been previously investigated. These findings prompted us to study the role of PANDAR in ccRCC.

In the present study, we found that PANDAR was significantly upregulated in ccRCC tissues compared to corresponding normal tissues. The upregulation of PANDAR was correlated with an advanced TNM stage and with lymph node involvement and distant metastasis. In vitro studies showed that PANDAR could regulate cell proliferation, migration and apoptosis. Furthermore, we demonstrated that PANDAR could modulate the anti-apoptotic proteins Bcl-2 and Mcl-1, as well as the PI3K/Akt/mTOR pathway.

## Methods

### Patient samples

This study was approved by the Human Ethics Committee of First Affiliated Hospital of Zhejiang University. ccRCC tissues and normal tissues were obtained from 62 patients who underwent nephrectomy or partial nephrectomy for ccRCC between 2012 and 2016. Written informed consent was obtained from all individual participants included in the study. None of the patients received local or systemic treatment before surgery. All tissues were washed with sterile PBS before being frozen in liquid nitrogen and then stored at −80 °C until analyzed. The pathological stage and grade were evaluated by an experienced pathologist.

### Cell culture

ccRCC cell lines 7860 and Caki-1 were obtained from the Shanghai bank of cell lines (Shanghai, China). The 7860 and Caki-1 cells were cultured in RPMI 1640 and DMEM medium, respectively, at 37 degree in a humidified atmosphere of 5% CO2.

### RNA extraction and quantitative real-time PCR

Total RNA was extracted using the Trizol reagent (Invitrogen, Carlsbad, CA, USA). cDNA was transcribed from total RNA using SuperScript III kit (Invitrogen). The primer sequences were as follows: PANDAR primers, forward: 5′- CTGTTAAGGTGGTGGCATTG-3′, reverse: 5′- GGAGGCTCATACTGGCTGAT-3′; and GAPDH primers, forward: 5′-CGCTCTCTGCTCCTCCTGTTC-3′, reverse: 5′- ATCCGTTGACTCCGACCTTCAC -3′. Quantitative real-time PCR was performed using the ABI PRISM 7000 Fluorescent Quantitative PCR System (Applied Biosystems, Foster City, CA, USA). The average value of each triplicate was used to calculate the relative amount of PANDAR using 2-ΔΔCt methods. Each sample was measured in triplicate.

### siRNA transfection

Small interfering RNA (siRNA) and nonspecific control siRNA or short hairpin RNA (shRNA) were synthesized (Sangon, Shanghai, China) and transfected into cells using Lipofectamine 3000 (Invitrogen, USA). The target sequence of si-PANDAR was 5′-GCAATCTACAACCTGTCTT-3′. The cells were cultured 24 h prior to transfection. Stably transfected cells were selected using G418 (Amresco, OH, USA).

### Western blotting

The lysates were resolved by 12% SDS-PAGE and then transferred to PVDF membranes. Primary antibodies against the following were used at 4 degree overnight: MMP-2 (Abcam, CA, USA); TIMP3 (Abcam, CA, USA); Cyclin D1 (Cellular Signaling Technology, MA, USA); Cyclin E1 (Cellular Signaling Technology, MA, USA); CDK4 (Cellular Signaling Technology, MA, USA); p21(Cellular Signaling Technology, MA, USA); Caspase-8 (Cellular Signaling Technology, MA, USA); Caspase-3 (Cellular Signaling Technology, MA, USA); cleaved PARP (Cellular Signaling Technology, MA, USA); Bcl-2 (Cellular Signaling Technology, MA, USA); Mcl-1 (Cellular Signaling Technology, MA, USA); Bax (Cellular Signaling Technology, MA, USA); p-PI3K (Cellular Signaling Technology, MA, USA); PI3K (Cellular Signaling Technology, MA, USA); p-Akt (T450) (Cellular Signaling Technology, MA, USA); p-Akt (S473) (Cellular Signaling Technology, MA, USA); Akt (Cellular Signaling Technology, MA, USA); mTOR (Cellular Signaling Technology, MA, USA) and GAPDH (Sigma, MO, USA). All chemicals were obtained from Sigma-Aldrich (MO, USA).

### Cell proliferation assay

Cell proliferation was assayed using a CCK-8 kit (Beyotime Biotech, China). Briefly, 2 × 103 cells/well were seeded in 96-well plates 24 h before the start of the experiment. The cells were then transfected with the corresponding si-RNA and cultured in medium. At 0, 24, 48, 72 and 96 h after transfection, 10 μl of CCK-8 (5 mg/ml) was added to each well and the cells were cultured for 1 h, and the absorbance at 450 nm was determined.

### Cell cycle analysis

Transfected cells were harvested after 48 h of incubation in 6-well plates. The cells were collected and fixed in ethanol. The cells were then washed with PBS and stained with propidium iodide (BD Bioscience) for 30 min in PBS supplemented with RNase at room temperature in the dark. The analysis was performed in triplicate, and the cell cycle distribution was evaluated using a flow cytometer (BD bioscience).

### Apoptosis assay

Transfected cells were harvested and double stained with an Annexin V Apoptosis Detection Kit (BD Bioscience). The cells were then analyzed using a flow cytometer (BD Bioscience).

### Colony formation assay

For the colony formation assay, 1000 cells were plated into 6-well plates and incubated in media. One week later, the cells were fixed and stained with 0.1% crystal violet and the visible colonies were counted.

### Cell invasion assays

The cell invasion assay was performed using 24-well insert transwell chambers (Corning, NY, USA). Cells were suspended in 200 μl of serum-free medium and were added to the upper chamber, and medium with 10% FBS was placed in the bottom chamber. The cells were then incubated for 48 h at 37 degree, and the cells on the upper surface were washed away, while the cells on the bottom surface were fixed with 20% methanol and stained with 0.1% crystal violet. The number of invaded cells was counted in five randomly selected fields using a microscope.

### Lentivirus generation and infection

Short hairpin RNA (shRNA) directed against human lncRNA PANDAR (sh-PANDAR) or scrambled oligonucleotides (negative control, sh-NC) were cloned into the LV-3 (pGLVH1/GFP + Puro) vector (a generous gift from Dr. Xinhua Lv, Zhejiang University). The 293 cells were co-transfected with Lenti-Pac HIV Expression Packaging Mix and the lentiviral vectors (Life Technologies Ltd., Carlsbad, CA, USA). After 48 h, lentiviral particles in the supernatant were harvested and filtered by centrifugation at 500 x g for 15 min.

### In vivo experiments

All of the experimental protocols were approved by the Animal Care and Use Committee of Quzhou People’s Hospital. The experiment is in compliance with the National Institutes of Health guide for the care and use of laboratory animals (NIH Publications No. 8023, revised 1978). Four-week-old BALB/c nude mice were randomly divided into two groups, with 4 mice in each group. The 7860 cells that were stably transfected with sh-NC or sh-PANDAR (5 × 10^6^ cells per mouse) were injected subcutaneously in the right flanks of mice. 27 days later, the mice were then sacrificed by cervical dislocation, and the tumors were removed and weighed.

### Statistical analysis

All statistical analyses were performed using SPSS 18.0 (IBM, Chicago, IL). A Paired Samples t-test was applied to analyze the difference in PANDAR expression between ccRCC tissues and adjacent normal tissues. The CCK-8 assay data were analyzed by ANOVA and Independent Samples t Test was used to analyze other data. Data from at least three independent experiments that were performed in triplicate are presented as the means ± standard deviations (SD). The significance of the differences between groups was estimated using Student’s t-test. OS rates were calculated using the Kaplan-Meier method with the log-rank test for comparisons. The Cox proportional hazards model was used in the multivariate and univariate analysis. Significance was defined as *P* < 0.05.

## Results

### PANDAR was upregulated in human ccRCC tissue and is associated with poor prognosis

**Fig. 1:**
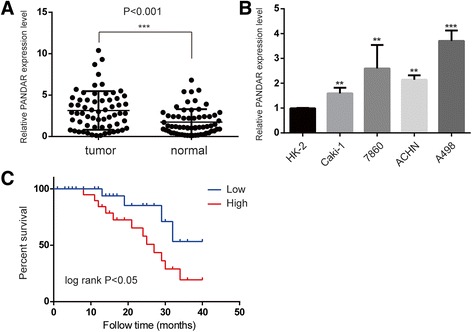
The relative expression levels of PANDAR in ccRCC tissues and cell lines. **a**. PANDAR expression levels were higher in ccRCC tissues than in pair-matched adjacent normal tissues. **b**. PANDAR was upregulated in ccRCC cell lines compared to that in the normal human proximal tubule epithelial cell line HK-2. **c**. Kaplan-Meier curves for overall survival of patients with ccRCC categorized according to PANDAR expression: significantly poorer overall survival was observed in patients with high PANDAR expression than those with low PANDAR expression (*P* < 0.05, log-rank test). Data represent mean ± SD, *P < 0.05; **P < 0.01; ***P < 0.001

To explore the role of PANDAR in ccRCC progression, the relative expression level of PANDAR was quantified by Real Time qPCR in 62 pairs of ccRCC and adjacent normal tissues; the results were normalized to GAPDH. As shown in Fig. 1a, the expression of PANDAR was significantly upregulated in tumor tissues when compared with pair-matched normal tissues (*P* < 0.001). The expression level of PANDAR was then evaluated in one normal kidney cell line (HK-2) and in four ccRCC cell lines (Caki-1, A498, ACHN and 7860). The data indicated that PANDAR expression was elevated in ccRCC cell lines compared with HK-2 cells (Fig. 1b). To assess the correlation of PANDAR expression with clinicopathologic data, the expression levels PANDAR in tumor tissues were categorized as low (*n* = 28) or high (*n* = 34) according to the median value of relative PANDAR expression (median expression value = 2.8). Correlation regression analysis indicated that PANDAR expression was positively correlated with the TNM stage (*P* = 0.029), lymph node metastases (*P* < 0.001) and distant metastases (*P* < 0.001). However, no association was found between the level of PANDAR expression and other parameters such as age and gender (Table 1).

**Table 1:** Clinicopathological features of patients with ccRCC

Variables	Number (%)	Expression of PANDAR		
		low	high	*P* value
Sex				
Male	39 (62.9%)	17	22	0.619
Female	23 (37.1%)	11	12	
Age, years				
≤ 60	18 (29%)	12	6	0.862
> 60	44 (71%)	16	28	
TNM stage				0.029
I	30 (48.4%)	17	13	
II-IV	32 (51.6%)	11	21	
Fuhrman grade				0.0511
G1-G2	25 (40.3%)	12	18	
G3-G4	37 (59.7%)	16	16	
Lymph node metastasis				<0.001
Negative	58 (93.5%)	28	30	
Positive	4 (6.5%)	0	4	
Distant metastasis				
Negative	59 (95.2%)	28	31	<0.001
Positive	3 (4.8%)	0	3	

We further analyzed whether the expression of PANDAR correlated with outcomes in ccRCC patients using Kaplan-Meier survival analysis. The Kaplan-Meier survival curve indicated that high levels of PANDAR expression are significant predictors of poor survival (*P* = 0.044) (Fig. 1c). As shown in Table 2, univariate analysis identified that PANDAR expression, the TNM stage, the Fuhrman grade, lymph node metastases and distant metastases are associated with the overall survival of patients with ccRCC (*P* < 0.05). In addition, multivariate analysis indicated that the expression of PANDAR was an independent prognostic factor for the overall survival of patients in line with the TNM stage, the Fuhrman grade, lymph node metastases and distant metastases. These data suggest that PANDAR may be involved in the progression and development of ccRCC.

**Table 2:** Univariate and multivariate analyses of clinicopathological factors for over survival

Variables	Univariate analysis			Multivariate analysis		
	HR	95% CI	*P* value	HR	95% CI	*P* value
PANDAR expression(High, Low)	1.74	1.07–5.66	0.002	1.13	0.98–5.12	0.014
TNM stage (I, II-IV)	4.77	1.77–9.72	0.001	3.88	1.22–8.77	0.003
Fuhrman grade (G1-G2,G3-G4)	2.36	0.89–10.78	0.001	2.09	0.66–9.33	0.022
Lymph node metastasis (yes, no)	4.47	2.13–8.44	0.011	3.73	1.87–7.11	0.001
Distant metastasis (yes,no)	6.77	3.11–6.88	0.008	5.21	2.09–5.74	0.004
Gender (male, female)	1.88	0.67–5.21	0.287			
Age (≤ 60, > 60)	1.08	1.81–3.66	0.332			

### Attenuated expression of PANDAR inhibits ccRCC cell proliferation and invasion

**Fig. 2:**
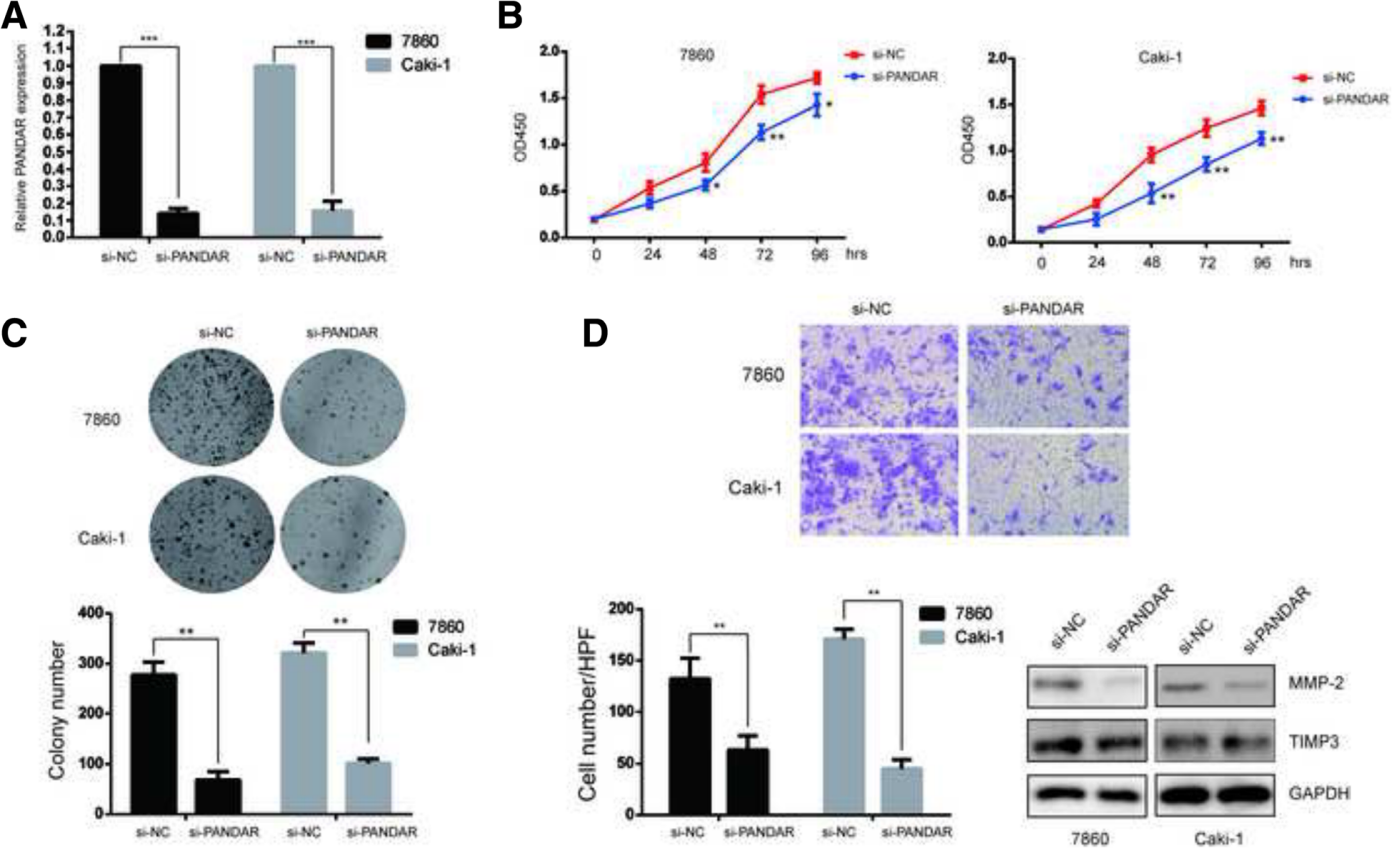
Knockdown of PANDAR inhibited ccRCC cell proliferation and invasion in vitro. **a**. PANDAR expression levels in 7860 and Caki cells transfected with si-NC or si-PANDAR were detected by qRT-PCR. **b**. The cell proliferation of 7860 and Caki cells transfected with si-NC or si-PANDAR was measured by CCK-8. **c**. Colony formation assays were performed to detect the proliferation of 7860 and Caki cells that were transfected with si-NC or si-PANDAR for 15 days. **d**. Transwell assays were performed to investigate the invasive ability of 7860 and Caki cells that were transfected with si-NC or si-PANDAR. The number inside the bars represent the relative ratio of invaded cells (normalized to the control). The lysates of 7860 and Caki cells were detected by Western blotting assays. Data represent mean ± S.D., (*n* = 3) *P < 0.05; **P < 0.01

To further confirm that the expression of PANDAR is positively associated with ccRCC progression, we used siRNA to silence the endogenous expression of PANDAR in 7860 and Caki-1 cells, which have the highest and the lowest levels of PANDAR, respectively. The qPCR results confirmed the efficiency of the siRNA in the two cell lines (Fig. 2a). As illustrated by CCK-8 assays, silencing of PANDAR markedly decreased the proliferation of 7860 and Caki-1 cells compared with the control groups (Fig. 2b). Furthermore, colony formation in PANDAR downregulated cells was significantly reduced as well (*P* < 0.01) (Fig. 2c).

**Fig. 3:**
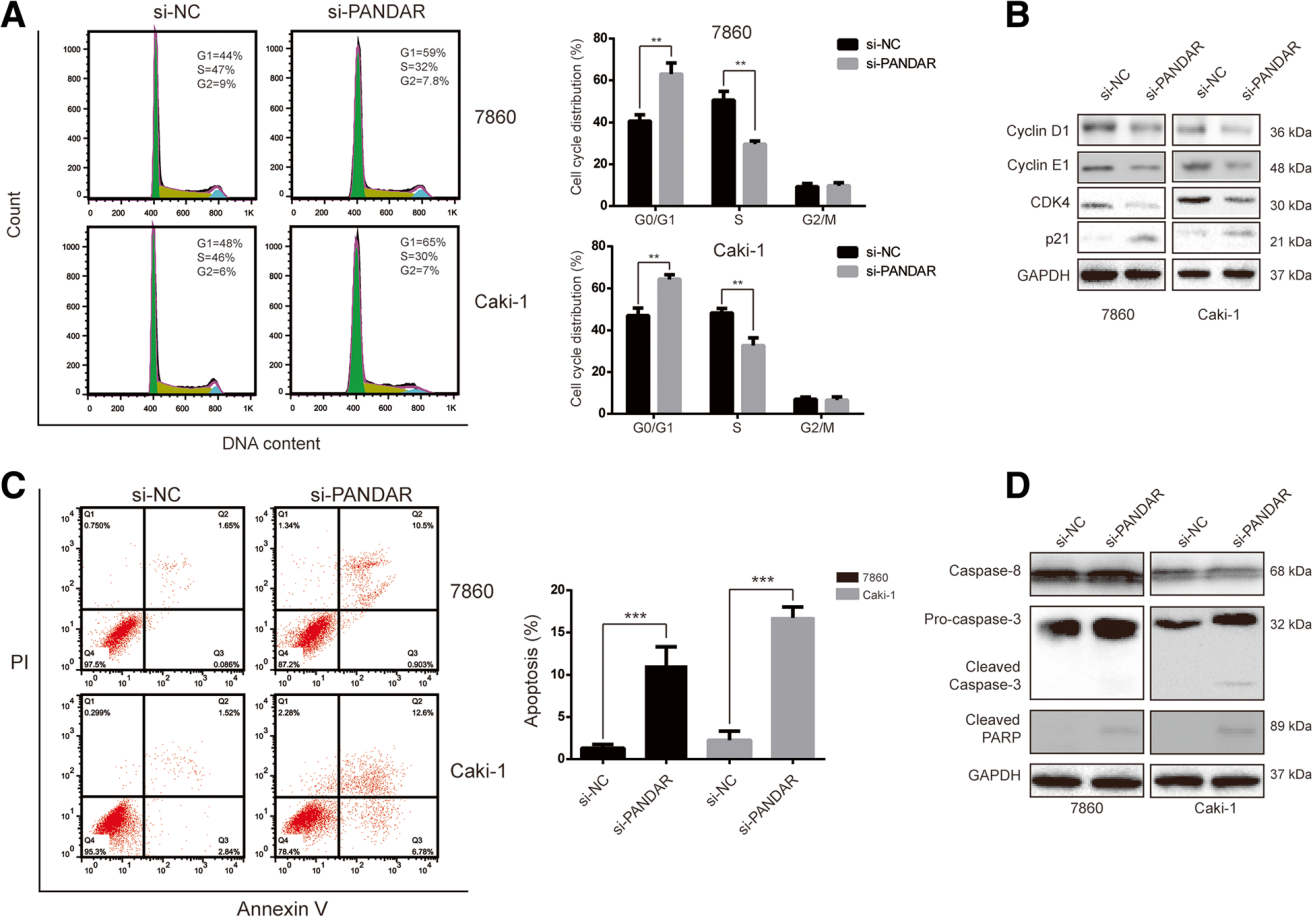
Silencing of PANDAR leads to cell arrest and apoptosis in ccRCC cells. **a**. Flow cytometry was used to analyze the cell cycle distribution of 7860 and Caki cells that were transfected with si-NC or si-PANDAR. **b**. Western blotting was used to detect the proteins involved in cell cycle distribution. **c**. Flow cytometry was used to analyze cell apoptosis of 7860 and Caki cells that were transfected with si-NC or si-PANDAR. **d**. Western blotting was used to detect the proteins involved in apoptosis. Data represent mean ± S.D., (*n* = 3) *P < 0.05; **P < 0.01

Cell invasion involves the migration of tumor cells into contiguous tissues and the dissolution of extracellular matrix proteins is an important aspect of cancer progression, we next evaluated the effects of PANDAR on cell invasion. The results of transwell assays are shown in Fig. 3d and indicate that silencing of PANDAR attenuated the invasive ability of 7860 and Caki-1 cells (*P* < 0.01). MMPs (Matrix metalloproteinases) and their inhibitors TIMPs (tissue inhibitors of matrix metalloproteinases) play a crucial role in cell migration and invasion [13]. To further explore the mechanism of PANDAR in suppressing ccRCC cell invasion, MMP2 and TIMP3 were examined using western blotting assays. The results demonstrated that the expression level of MMP2 was significantly reduced after the knockdown of PANDAR (Fig. 2d). However, the expression level of TIMP3 was not affected (Fig. 2d).

### Downregulation of PANDAR induces cell cycle arrest and apoptosis in RCC cells

To determine whether the proliferative effects of PANDAR on RCC cells resulted from an alteration of the cell cycle or apoptosis, a flow cytometry analysis was performed. As shown in Fig. 3a, transfection of siRNA against PANDAR caused cell cycle arrest at the G0/G1 phase in both cell lines. Simultaneously, the proportion of cells in the S phase decreased. To further explore the molecular mechanisms responsible for the induced cell cycle arrest in the G0/G1 phase that was caused by the downregulation of PANDAR, the expression levels of G0/G1 regulatory proteins were determined using a Western blotting assay. We observed that the expression levels of cyclin D1, cyclin E1 and CDK4 were remarkably reduced, while p21 was significantly upregulated after the transfection of siRNA against PANDAR for 24 h in both cell lines (Fig. 3b). All of these data indicated that repression of PANDAR could induce a G0/G1 arrest in RCC cells via altering the expression of key proteins in the Cyclin-CDKs signal pathway.

Next, we asked whether apoptosis was a contributing factor to cell growth inhibition. First we transfected the cells with si-PANDAR or si-NC for 24 h, and then stained the cells with Annexin V/PI and evaluated the cells using flow cytometry. As shown in Fig. 3c, there is a significant increase in apoptotic cells in the si-PANDAR-treated cells compared with the si-NC-treated groups. The involvement of apoptosis was further confirmed using Western blotting to check the changes in apoptosis-associated proteins. Cleaved caspase-3 and PARP were detected after the downregulation of PANDAR, whereas caspase-8 protein levels were unchanged in both 7860 and Caki-1 cells (Fig. 3d).

### Attenuated expression of PANDAR affected the expression of Bcl-2 family members and inhibited the PI3K/Akt/mTOR pathway

**Fig. 4:**
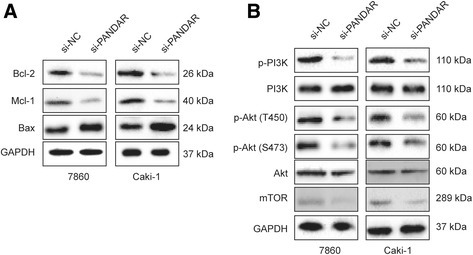
PANDAR regulates Bcl-2 family proteins and the PI3K/Akt/mTOR signaling pathway in ccRCC cells. **a** and **b**. The expression levels of the indicated proteins were detected by Western blotting in 7860 and Caki cells that were transfected with si-NC or si-PANDAR

Due to the cleavage of caspase-3 but not caspase-8 was observed, we hypothesized that PANDAR may affect cellular apoptosis through the intrinsic apoptotic pathway. Bcl-2 family proteins play an important role in the process of cell apoptosis. We determined the expression of Bcl-2 family members using Western blotting assays. As shown in Fig. 4a, the levels of Bcl-2 and Mcl-1 were downregulated, while Bax was upregulated after the silencing of PANDAR.

Many studies have indicated that the PI3K/Akt/mTOR pathway is involved in cell proliferation and apoptosis in various cancer cells [14, 15]. Therefore, we asked whether the PI3K/Akt/mTOR pathway was affected after the silencing of PANDAR. As shown in Fig. 4b, after transfection with si-PANDAR for 24 h, Akt phosphorylation at Thr450 and Ser473 were inhibited remarkably, while the total protein level of Akt remained constant in both cell lines. In addition, mTOR, a well-known downstream target of Akt, was downregulated (Fig. 4b). These data suggested that the PI3K/Akt/mTOR pathway played an important role in PANDAR-mediated cell proliferation and apoptosis.

### PANDAR regulated RCC growth and apoptosis in vivo

**Fig. 5:**
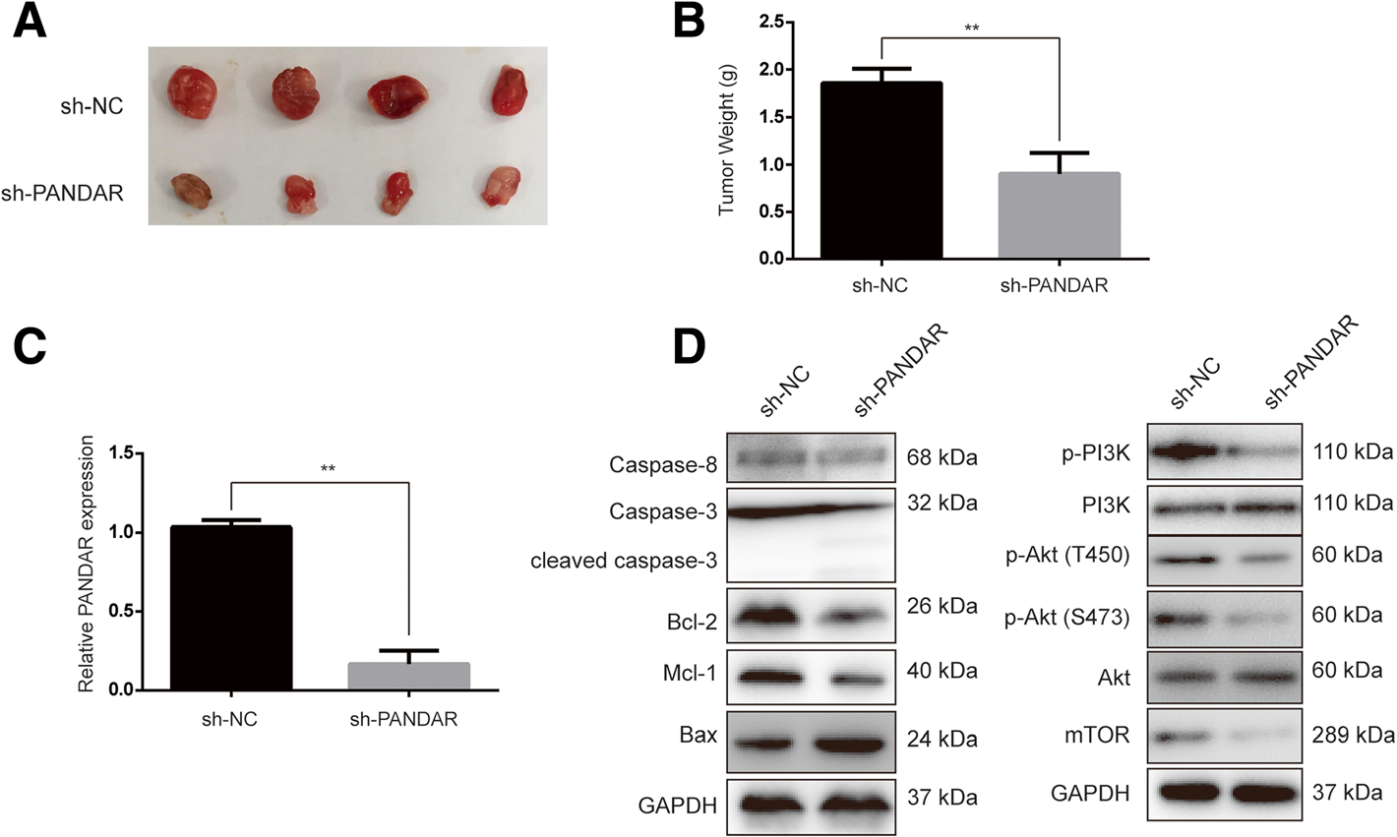
Silencing of PANDAR regulated RCC growth in vivo. **a**. Tumors from 7860 cells that were stably transfected with sh-NC or sh-PANDAR. **b**. A comparison of tumor weights between sh-NC and sh-PANDAR groups. **c**. qRT-PCR was performed to detect PANDAR expression levels in tumors from 7860 cells that were stably transfected with sh-NC or sh-PANDAR. **d**. The indicated proteins were determined in tumors from 7860 cells that were stably transfected with sh-NC or sh-PANDAR by Western blotting. sh-NC, short hairpin RNA of negative control; sh-PANDAR, short hairpin RNA for PANDAR. Data represent mean ± S.D., (*n* = 3) *P < 0.05; **P < 0.01

To explore whether PANDAR affected tumorigenesis of RCC, 7860 cells that were stably transfected with sh-NC or sh-PANDAR were injected into nude mice which were sacrificed 27 days later. As shown in Fig. 5a and b, the tumor weights for the sh-PANDAR group were remarkably lower than those in the sh-NC group. Furthermore, qRT-PCR demonstrated that the expression of PANDAR in the sh-PANDAR group was lower than its expression in the sh-NC group (Fig. 5c). Consistent with in vitro experiments, when compared with the sh-NC group, tumors in the sh-PANDAR group exhibited upregulated expression of Bax, cleaved caspase-3 and reduced expression of Bcl-2, Mcl-1 (Fig. 5c). In addition, the PI3K/Akt signaling pathway was inhibited after the silencing of PANDAR, which is in accordance with in vitro results as well. Those data further confirmed the function of PANDAR in RCC tumorigenesis.

## Discussion

ccRCC is one of the most deadly genitourinary malignancies. The prognosis for renal cell carcinoma is quite poor because most ccRCC patients are diagnosed at a later stage when treatment is not effective [16]. Therefore, the identification of novel prognostic biomarkers and therapeutic targets may have an enormous potential to improve the outcomes of ccRCC.

It is now estimated that only 2% of the human genome can be translated into proteins, whereas 60–70% of genome is transcribed into non-coding RNAs (ncRNAs) [17]. Among these ncRNAs, lncRNAs, which are longer than 200 nucleotides, are important new members of ncRNAs [4]. In recent years, lncRNAs have received great attentions because lncRNAs are involved in tumor development and therefore possess the potential to be biomarkers and prognosis factors [18]. Research regarding the functions of lncRNAs in ccRCC is diverse. For example, lncRNA ZNF180–2 and MALAT1 were found to be upregulated in ccRCC tissues and are associated with poor prognosis [19, 20]. Conversely, the lncRNA CADM1-AS1 functions as a tumor suppressor in ccRCC [21]. Moreover, it has been reported that several novel lncRNAs were dysregulated in ccRCC, but there is no correlation between lncRNA expression and the clinicopathological features of ccRCC [22].

PANDAR is a newly identified lncRNA that is localized at chromosome 6 and has a length of 1506 nucleotide [8]. The role of PANDAR in tumorigenesis is still controversial. For instance, PANDAR was downregulated in non-small cell lung cancer (NSCLC), and the low level of PANDAR indicated a poor prognosis [11]. In contrast, PANDAR was significantly upregulated in bladder cancer [23]. Currently, there are no reports on the clinical relevance of PANDAR to ccRCC. In the present study, we sought to determine whether there was any difference in the expression of PANDAR between ccRCC tissues and adjacent normal tissues. We found that the expression levels of PANDAR in ccRCC tissues were significantly higher. In addition, we demonstrated that increased PANDAR expression was positively correlated with an advanced TNM stage, lymph node metastases, distant metastases and poor prognosis. Moreover, the expression of PANDAR is higher in ccRCC cell lines than in normal renal cell lines. These results suggest that PANDAR may play a role in the development of ccRCC. To understand the biological functions of PANDAR in ccRCC, we evaluated cell proliferation, the cell cycle, apoptosis and invasion after silencing of PANDAR in 7860 and Caki-1 cells. After the downregulation of PANDAR, both cell lines exhibited a marked decrease of cell proliferation and invasion. We also observed a cell cycle arrest in the G0/G1 phase, which is similar to the findings of Sang et al. who demonstrated that the silencing of PANDAR caused a G0/G1 phase arrest in breast cancer [24]. In addition, we observed that the knockdown of PANDAR led to greater apoptosis in both cell lines. To unveil the potential mechanisms by which PANDAR promotes proliferation, invasion and the inhibition of apoptosis, we measured proteins that are involved in these biological processes.

The G1-phase-related Cyclin-CDK complex is inhibited to promote cell cycle arrest [25]. Here, our finding of a decrease in cyclin D1, cyclin E1 and CDK4 in both cells after silencing of PANDAR suggests the disruption of the uncontrolled cell cycle progression of 7860 and Caki-1 cells. We also observed that MMP-2 and TIMP-3 were downregulated after the knockdown of PANDAR, and MMP-2 and TIMP-3 have been implicated in the regulation of the metabolism of the extracellular matrix, tumor progression and metastasis [26]. Thus, our data suggests that PANDAR might promote cell invasion by regulating the expression of MMP-2 gene.

On the other hand, flow cytometry analysis demonstrated that the downregulation of PANDAR resulted in the induction of apoptosis. Apoptosis is one form of programmed cell death and is considered to be a protective mechanism that eliminates mutated neoplastic cells [27]. Apoptosis is tightly regulated by pro-apoptotic and anti-apoptotic proteins such as caspases and Bcl-2 family proteins. We found that caspase-3 but not caspase-8 protein was cleaved after the silencing of PANDAR. Because Bcl-2 family proteins (Bcl-2, Mcl-1 and Bad) could affect the activation of caspase-3, we asked whether the activation of caspase-3 by the downregulation of PANDAR was due to the alteration of Bcl-2 proteins. We found that silencing of PANDAR could inhibit Bcl-2 and Mcl-1 while enhancing the expression of Bax. Therefore, PANDAR may at least affect ccRCC cell apoptosis through modulation of Bcl-2 family proteins. The present study also examined the signaling pathway that is possibly involved in apoptosis, and it was found that the silencing of PANDAR inhibited the expression of mTOR and the phosphorylation of PI3K and Akt. These results were also supported by the in vivo experiments. Taken together, these results indicate that PANDAR may promote cell proliferation via cell cycle arrest and apoptosis partly through the PI3K/Akt/mTOR pathway.

However, it is worth noting some of the limitations in the present study. First, the function of PANDAR was investigated using RNA interference, and there is a lack of gain-of-function approach, such as overexpression of PANDAR. Second, although we found that PANDAR is upregulated in ccRCC, the mechanisms underlying this dysregulation remains elusive.

## Conclusions

In conclusion, we have demonstrated that lncRNA PANDAR is upregulated in ccRCC tissues and is significantly associated with advanced tumor progression. Moreover, the expression of PANDAR was demonstrated to be an independent marker for predicting the clinical outcome of ccRCC patients. Our results indicate that PANDAR could function as a tumor-promoting gene by regulating the G1/S transition and by promoting tumor invasion. In addition, we demonstrated that PANDAR-mediated cell apoptosis is at least partially mediated via the regulation of Bcl-2 family members and the PI3K/Akt/mTOR pathway. Taken together, these data suggest that PANDAR is a promising biomarker and therapeutic target for the treatment of ccRCC.

## Abbreviations

ccRCC: Clear cell renal cell carcinoma
lncRNA: Long non-coding RNA
mTOR: The mammalian target of rapamycin
MTT: Methyl thiazolyl diphenyl tetrazolium bromide
PANDAR: Promoter of CDKN1A antisense DNA damage activated RNA
PARP-1: Poly(ADP-ribose) polymerase-1
PI: Propidium iodide
PI3K: Phosphatidylinositol 3-kinase
